# Effect of Qing Chang oral liquid on the treatment of artificially infected chicken coccidiosis and the cellular immunity

**DOI:** 10.1002/vms3.922

**Published:** 2022-09-16

**Authors:** Yan Zhi‐qiang, Chen Qian‐lin, Fu Li‐zhi, Fu Wen‐gui, Zheng Hua, Tang Hong‐mei, Zhai Shao‐qin, Chen Chun‐lin

**Affiliations:** ^1^ Chongqing Academy of Animal Sciences Chongqing China; ^2^ College of Veterinary Medicine Xinjiang Agriculture University Xinjiang Urumqi China; ^3^ Chongqing Research Center of Veterinary Biological Products Engineering and Technology Chongqing China

**Keywords:** chicken coccidiosis, cytokines, Qing Chang oral liquid, T‐lymphocyte subsets

## Abstract

**Background:**

Qing Chang oral liquid (QOL) is a veterinary drug, which mainly composed of *Artemisiae annuae herba*, *Dichroae radix*, *Agrimonia pilosa* and *Sanguisorbae radix*.

**Objectives:**

This study aims to explore the effect of Qing Chang Oral Liquid (QOL) on the treatment effect of artificially infected chicken coccidiosis and to the cellular immunity.

**Methods:**

Healthy Roman chickens were randomly divided into five groups: blank group, model group, QOL high‐, medium‐ and low‐dose groups. All the groups were orally administered with 1 × 10^4^ sporulated oocysts (except the blank group). After 5 days of oral administration, the high‐, medium‐ and low‐dose groups of QOL were added to the drinking water at 2.4, 1.8 and 1.2 ml/kg, respectively. The blank and model groups were fed normally, and this experiment lasted for 7 days. The clinical signs were observed, and the relative weight gain, survival rate, cecum lesion score and oocyst value were measured to evaluate the effect of QOL. Meanwhile, the peripheral blood T‐lymphocyte subsets and cecal IL‐2, IL‐17, IFN‐γ mRNA expression were detected by flow cytometry and fluorescent quantitative PCR.

**Results:**

The chickens in the model group were in poor mental state, gathered together, had loose stools or bloody stools and had less food intake and less exercise. The chicken mental state improved, the food intake and drinking water increased, and the faeces are normal in the high‐, medium‐ and low‐dose groups, especially in the high‐dose group, the anti‐coccidial indexes were up to 173.08. No significant differences were observed (*p* > 0.05) in the peripheral blood CD3^+^, CD3^+^CD4^+^ between the experimental groups. Compared with the blank group, there were different degrees of increase in each dose drug group of the cecal IFN‐γ, IL‐2 and IL‐17 mRNA expression, but the high‐dose group was significantly reduced compared with the model group (*p* < 0.01), but there was no significant difference (*p* > 0.05).

**Conclusions:**

This study suggests that QOL has positive anti‐coccidial effect but has no obvious effect on the cellular immunity.

## Graphical abstract

Qing Chang oral liquid (QOL) is a veterinary drug, which mainly composed of *Artemisiae annuae herba*, *Dichroae radix*, *Agrimonia pilosa* and *Sanguisorbae radix* through its anti‐coccidial and cellular immunity studies. This study suggests that QOL has positive anti‐coccidial effect but has no obvious effect on the cellular immunity.

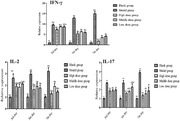

## 1 INTRODUCTION

Avian coccidiosis, such as Eimeria, is an intestinal infection caused by eimerian parasites, is an economically important disease worldwide (Williams, 2002; Wang, 2019) and is responsible for major economic losses in poultry industry by increasing mortality and reducing growth rates (Guo et al., 2007; Abbas et al., 2017). Current means of control the coccidiosis mainly rely on the use of coccidiostats, such as sulfa drugs and diktrillin. However, these are associated with irreversible drawbacks like drug resistance, drug residues, high production cost, variable efficacy between batches (Shah et al., 2010). Therefore, alternate approaches and the research and development of new, safe and efficient coccidiosis drugs are urgently needed (Bi et al., 2018; Tumenbayaer et al., 2019).<COMP: Please set reference citations as per the journal style, that is, in alphabetical order.>

Cellular immunity is a protective immune process that involves the activation of phagocytes, antigen‐sensitized cytotoxic T cells and the release of cytokines and chemokines in response to antigen (Gurunathan et al., 1998). Furthermore, the studies clearly indicated an important role of various effector mechanisms involving T lymphocytes, macrophages, natural killer (NK) cells and cytokines in resistance to coccidiosis (Lillehoj, 1998).

Traditional Chinese Medicines usually use active ingredients in plants, which has been proved to be effective in many diseases treatment. The use of herbal medicines has been becoming increasingly popular as alternative and/or complementary therapies around the world (De Smet, 2002). Qing Chang oral liquid (QOL) is a traditional Chinese veterinary drug, which is mainly composed of *Artemisiae annuae herba*, *Dichroae radix*, *Agrimonia pilosa* and *Sanguisorbae radix*. Modern pharmacological studies have found that the main anti‐coccidial components in *Artemisia annua* and Changshan are artemisinin and Changshan alkaloid (Jiao et al., 2018; Yu, 2019). These four ingredients of plant traditional Chinese Medicine are proved to have anti‐inflammatory or anti‐coccidiosis effect. Our preliminary study found that the QOL has positive therapeutic effect on coccidiosis of chicken (Yan et al., 2019). However, the mechanism of anti‐coccidiosis is still unclear. The objective of the present experiment was to investigate the effect of QOL, which was isolated from different Chinese medicinal plants, on the clinical signs including the relative weight gain, survival rate, cecum lesion score and oocyst value to evaluate the effect of QOL and on the cellular immunity after coccidiosis infection.

## 2 MATERIALS AND METHODS

### 2.1 Experiment drugs and preparation

The component of the Qing Chang Oral Liquid (QOL) was *Artemisiae annuae herba*, *Dichroae radix*, *Agrimonia pilosa* and *Sanguisorbae radix*; they were crushed and mixed according to the ratio of 5:2:2:1. The proportion of each drug is based on the formulation theory of Chinese veterinary medicine monarch, and the results are verified by clinical screening. Decocted with water for three times, each time for an hour, and then combined the above three times water and concentrated to 1 ml liquid containing 1 g of raw medicine, artemisinin content shall not be less than 3 mg/ml, and the content of Changshan alkaloid shall not be less than 80 μg/ml of solution. *Artemisiae annuae herba* (2020.12.03), *Dichroae radix* (2020.10.13), *Agrimonia pilosa* (2021.01.05) and *Sanguisorbae radix* (2020.11.11), all the ingredients of traditional Chinese Medicine were purchased from Chongqing Huasen Pharmacy. The quality of the medicinal materials conforms to the veterinary drug code of the People ’s Republic of China, Volume II, 2015 Edition.

### 2.2 Laboratory animals and the preparation of the *Eimeria*

One hundred and fifty 1‐day‐old healthy Roman Pink chicks were purchased from Yongchuan Yiping chicken farm in Chongqing. The chickens in each group were fed in experimental cages, were in routine feeding management, fed freely and given water ad libitum until they were 16 days old.

Sixteen‐day‐old chickens were randomly divided into five groups: control group, model group, QOL high group, QOL medium group and QOL low group, with five replicates (6 broilers in each replicate). Model replication was performed by Yan et al. (2020); 1 × 10^4^ spore oocysts were fed in all groups except the control group, and the same volume of normal saline was fed to the control group for 5 days.

The clinical dosage is calculated by referring to the dose conversion of chicken coccidia powder (10–20 g/kg feed, the average weight of the test chicken is 200 g, the average daily intake is 30 g, i.e. 0.3–0.6 g, the average weight is 1 kg, the dose of the test chicken is 1.5–3.0 g and the specification of Qing Chang oral liquid in this study is 1 ml, including 1 g of the original drug) in the veterinary medicine code of the People's Republic of China 2015. The doses of high, medium and low groups were 2.4, 1.8 and 1.2 ml/kg, and added to the drinking water, once a day, for 7 days, the control group and the model group were feed normally.

The ova of *Eimeria tenella* were isolated from the cecum of the chickens naturally infected with coccidiosis and preserved by Chongqing Academy of Animal Sciences.

### 2.3 Efficacy of QOL against coccidiosis

#### 2.3.1 Relative weight gain rate and survival rate

Chickens in each group were weighed and recorded before and at the end of the study. The average weight gain and survival rate were calculated of the death chickens infected by coccidium. Relative weight gain rate (%) = average weight gain of sick chickens in each group/average weight gain of health chickens in control group × 100%; survival rate (%) = the number of surviving chickens/the number of chickens × 100%

#### 2.3.2 Count of the faecal coccidioides and cecal lesion score

Seven days after the treatment of the QOL, the faecal were collected of each chicken, and the parasite eggs were counted and the oocyst ratio was calculated according to the national standard method. At the end of experiment, the chicken were euthanized for the cecum, the cecum lesion was scored and the cecum lesion rate was calculated. Oocyst rate (%) = the average number of the oocyst in each group/ average number of the oocyst in model group × 100%. Lesion rate (%) = the total of lesion score in one group/the number of chicken in one group × 100%.

#### 2.3.3 Efficacy evaluation of QOL

The anti‐coccidial index of the QOL was calculated according to relative weight gain rate, survival rate, lesion rate and the oocyst rate of in each group. Anti‐coccidial index (ACI) = (relative weight gain rate + survival rate) – (lesion rate + oocyst rate).

The pharmacodynamic standards were as follows: poor effect, ACI is less than 120; medium effect, ACI is between 120 and 160; good effect, ACI is 160 and 180; excellent effect, ACI is more than 180.

### 2.4 Effect of QOL on cellular immunity of chickens artificially infected with coccidiosis

#### 2.4.1 Effect of QOL on T‐lymphocyte subsets in peripheral blood

The blood was collected from the wing vein of the chicken and added to the flow tube containing lymphocyte isolation solution, and then centrifuged and the interlayer lymphocytes were taken and transferred to a new flow tube, washed with PBS solution and centrifuged again, and at last the concentration of the cells was adjusted to 10^6^/ml. 500 μl cell suspension was taken in new flow tube and added mouse anti‐chicken CD3‐SPRD, mouse anti‐chicken CD4‐FITC and mouse anti‐chicken CD8α‐PE antibody, respectively. After dyeing, the supernatant was discarded by centrifugation, added 500 μl PBS solution to the flow tube, and detected by CytoFLEX flow cytometry from Backman (FACS of Becton Dickinson Company) in 1 h. Kaluza 2.1 software was used to experiment data analysis.

#### 2.4.2 Effect of QOL on mRNA expression of cytokines

Cecum was collected and stored in liquid nitrogen. Real‐time fluorescence quantitative PCR method (RT‐FQ PCR) (LightCycler 96, Roche) was used to test mRNA expression of the IFN‐γ, IL‐2 and IL‐17. The primer sequence information is shown in Table 1.

**TABLE 1:** Primer sequence

Gene	Primer name	Primer sequence(5'→3')	Amplification size (bp)
IFN‐γ	F	AACCTTCCTGATGGCGTGAA	137
	R	AAACTCGGAGGATCCACCAG	
IL‐2	F	TCTTTGGCTGTATTTCGGTAGC	153
	R	TTGGTGTGTAGAGCTCGAGAT	
IL‐17	F	GAGCCAGGCCGTAACGATT	104
	R	GGGGCTGCATTCCTAATTCA	
GAPDH	F	CAGAACATCATCCCAGCGTC	133

### 2.5 Statistical analyses

Statistical analysis of the results was conducted by IBM SPSS Statistics 21.0 (SPSS Inc., 181 Chicago, IL, USA), and the results were expressed as mean ± standard deviation (M ± SD). *p* Values less than 0.05 were considered as statistically significant.

## 3 RESULTS

### 3.1 Effect of QOL on clinical signs of artificially infected with chicken coccidiosis

There were obvious changes of the chicken in the model group and QOL group, they presented a good mental state, food intake and drinking water and faeces were normal after feeding the QOL for 3 days. After the 5 days, the chickens in the above groups were in poor mental state, were fearful of cold, clustered, began to appear apathetic, and significantly reduced intake of food and drinking water. Additionally, they lose sticky faeces with blood, messy and matte coat. After 7 days of administration, the mental state of chickens was improved, faeces were formed, there was no blood in the faeces and the intake of food and drinking water became normal.

### 3.2 Therapeutic effect of QOL on artificially infected with chicken coccidiosis

As shown in Table 2, the comprehensive survival rate, relative growth rate, oocyst value and lesion value were calculated. The anti‐coccidiosis index of the high‐, medium‐ and low‐QOL groups was 173.08, 162.63 and 150.12, respectively. According to the pharmacodynamics criterion, the group with high dose of QOL had good curative effect.

**TABLE 2:** Treatment effect of QOL on artificially infected chicken coccidiosis

Groups	Relative weight gain rate (%)	Survival rate (%)	Lesion value	Oocyst value	ACI
Control group	100	100	0	0	200
Model group	51.46	100	18.00	40	93.46
High group	80.08	100	7.00	0	173.08
Medium group	75.87	100	8.24	5	162.63
Low group	68.93	100	5.81	5	150.12

### 3.3 The Effect of QOL on T‐lymphocyte subsets in peripheral blood

There was no significantly difference in the percentages of CD3^+^, CD3^+^CD^4+^ and CD3^+^CD8^+^ in each experimental group (Table 3) at 3, 5 and 7 days after treatment with the QOL.

**TABLE 3:** Effect of QOL on peripheral blood T‐cell subsets in test chickens (%)

	3 days			5 days			7 days		
Groups	CD3^+^	CD3^+^CD4^+^	CD3^+^CD8^+^	CD3^+^	CD3^+^CD4^+^	CD3^+^CD8^+^	CD3^+^	CD3^+^CD4^+^	CD3^+^CD8^+^
Control	7.76 ± 0.79	5.19 ± 0.57	1.63 ± 0.34	8.55 ± 0.56	5.26 ± 0.29	1.66 ± 0.32	10.44 ± 1.10	5.81 ± 0.47	2.23 ± 0.39
Model	8.49 ± 0.74	5.24 ± 0.62	1.64 ± 0.29	8.94 ± 0.32	5.85 ± 0.37	1.36 ± 0.26	8.43 ± 1.12	4.68 ± 0.56	2.02 ± 0.29
High	8.48 ± 0.57	5.36 ± 0.41	1.94 ± 0.41	8.41 ± 0.93	5.53 ± 0.45	1.49 ± 0.33	9.39 ± 1.01	5.41 ± 0.76	2.18 ± 0.21
Medium	7.99 ± 0.81	5.00 ± 0.40	1.14 ± 0.39	8.78 ± 0.72	6.07 ± 0.45	1.34 ± 0.18	9.43 ± 0.76	5.28 ± 0.58	2.38 ± 0.16
Low	7.84 ± 0.80	4.89 ± 0.50	1.51 ± 0.43	9.18 ± 0.68	6.33 ± 0.54	1.32 ± 0.24	10.02 ± 1.02	4.73 ± 0.46	2.22 ± 0.22

*Note*: Means in a column with different letters are different, a capital letter means (*p* < 0.01), a small letter means (*p* < 0.05), and there is no difference (*p* > 0.05) for means in a row without letter or with the same letter.

### 3.4 Effect of QOL on mRNA expression of immune‐related genes in chicken cells

**FIGURE 1:**
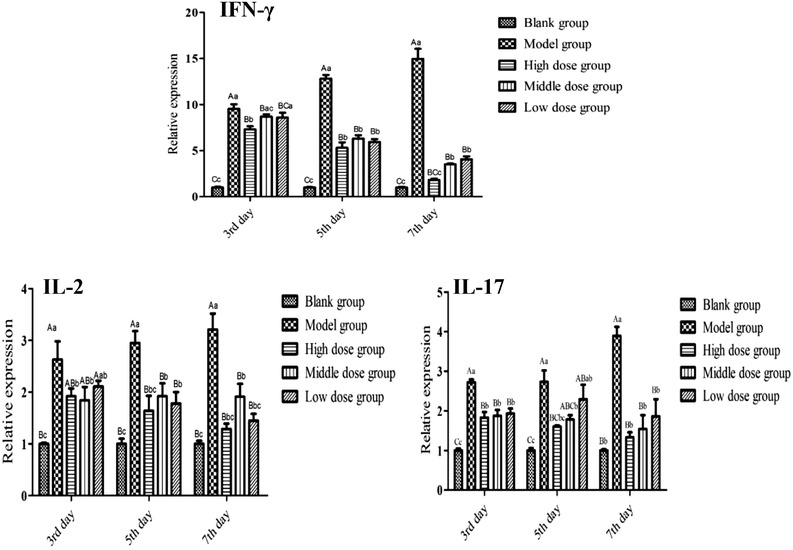
Effect of QOL on the expression level of IFN‐γ, IL‐2 and IL‐17 mRNA in the cecum

As showed in Figure 1, compared with the model group, the expression of IFN‐γ mRNA in each QOL group decreased significantly (*p* < 0.01) at 3, 5 and 7 days, and there was no significantly difference between the high QOL group and the control group (*p* > 0.05) at 7 days; IL‐2 mRNA expression in each QOL group was significantly reduced (*p* < 0.01), and there was no significant difference between the high and the control group (*p* > 0.05) at 5 and 7 days; IL‐17 mRNA expression level of each QOL group decreased significant (*p* < 0.01) at 3 days and in the high group at 5 and 7 days compared with the model group; and there was no significant difference between the high and the control group (*p* > 0.05).

## 4 DISCUSSION

According to the theory of veterinary medicine and the principle of clinical syndrome differentiation, avian coccidiosis belongs to the category of entomogenous accumulation, with dampness and heat gathering in the large intestine and is suitable for insect repellent and debulking and cooling blood and diarrhoea (Jiao et al., 2018). QOL is a Chinese veterinary medicine with good therapeutic effect on avian coccidiosis (Xing et al., 2017) which is mainly composed of *Artemisiae annuae herba*, *Dichroae radix*, *Agrimonia pilosa* and *Sanguisorbae radix*. It has been reported that *Artemisiae annuae herba* can affect the mitochondrial membrane potential and apoptotic protein activity of coccidioides, thus achieving the purpose of anti‐coccidiosis (Zhang et al., 2018; Ma et al., 2014). *Dichroae radix* can significantly inhibit the first‐ and second‐generation schizozoites, thus treating coccidiosis (Wang et al., 2018). *Agrimonia pilosa* has antioxidant activity (Set et al., 2017) or anti‐inflammatory and anti‐allergic effects (Kim et al., 2012). *Sanguisorbae radix* has nitric oxide production‐suppressing activity (Yokozawa et al., 2000) and antioxidant activity (Rhim, 2013). In this study, the mixture of them (in the form of QOL) was used to evaluate the effect on coccidiosis infection.

The cecum of chicken is the target organ where could be invaded by eimerian coccidioides. Coccidioides produce a large number of splinters to invade and damage the intestinal epithelial cells at the reproductive stage of fission (Macdonald et al., 2018; Srinivasn et al., 2019) and affect the digestion and absorption function of the intestine, which finally inhibit the growth of the chicken. Oocysts per gram (OPG) can indirectly reflect the ability of coccidioides oocysts to reproduce in animal bodies, and the degree of cecal lesions, relative weight gain rate and the amount of OPG can be used as important indicators for the evaluation of drug efficacy (Zou et al., 2019; Cha et al., 2018). In this study, we found that high dose of QOL could decrease the lesion value and oocyst value of the sick chickens significantly and increase the relative weight gain rate. The anti‐coccidiosis index of the QOL was 173.08, which has positive anti‐coccidiosis effect.

Avian coccidioides is an endoparasite that has closed relationship with the cellular immunity mediated by T lymphocytes and plays an important role in host resistance to coccidioides. Mature T cells are divided into CD4^+^ T cells and CD8^+^T cells according to the characteristic molecules on the cell surface. It has been found that CD4^+^ T lymphocytes, after being stimulated by antigen, promote the activity of natural killer cells and enhance the cytotoxic effect of T cells and play an important role in fighting coccidiosis primary infection (Hurtgen & Hung, 2017). CD8^+^ T lymphocytes play vital roles in virus immediate‐early antigens mediate protective immunity (Reddehase et al., 1987). It has been reported that the CD4^+^ T lymphocytes play key roles in the control of primary infections with Eimeria spp., and CD8^+^ T lymphocytes participate in the expression of resistance to reinfection (Rose et al., 1992). In the present study, it was found that after administration of QOL when infected with *Eimeria* spp., the percentage of CD4^+^ T cells in the high and medium dose QOL group was increased in comparison with the model group, while the percentage of CD8^+^ T cells showed no significant change. It is speculated that QOL can promote the proliferation of CD4^+^ T cells to a certain extent and then enhance the cellular immune function of the chicken. The anti‐coccidian effect may be achieved by the effective component of QOL, which is the alkaloid of Changshan, acting on the developing sporozoites in the first‐generation schizoites and the second‐generation schizoites of coccidian (Chen et al., 2012).

Cytokines are low molecular soluble proteins induced by immunogen, mitogen or other stimulants, which can regulate the immune function, remove antigens and repair damaged tissues and so on. In this study, IFN‐γ, IL‐2 and IL‐17 were chosen for detection; these cytokines play important roles in resistance to coccidiosis infection. IFN‐γ is produced by CD4^+^ Th1 and activated NK cells, mediating the cellular immune response and inhibiting the reproduction of coccidioides spore to resist coccidiosis infection, which is considered to be the most important cytokine in the resistance to coccidiosis infection (Hung et al., 2016). IL‐2 is mainly produced by Th1 and NK cells, activates helper T cells and cytotoxic T cells, stimulates T‐cell proliferation and cytotoxicity and plays an important role in coccidiosis infection (Lillehoj et al., 2005; Wang et al., 2020). IL‐17, produced mainly by Th17 cells, has both anti‐infective and pro‐inflammatory properties and plays a role in fighting coccidiosis infection (Min et al., 2013). In this study, it was found that the expression of IFN‐γ, IL‐2 and IL‐17 mRNA in the cecum of QOL treatment was decreased when compared with the model group, and it was extremely significant decrease in the high‐dose group, while there was no significant difference when compared with the blank group. The results suggested that QOL can significantly decrease the oocyst value of coccidiosis chicken and thus decrease the degree of coccidiosis infection. This may be the main reason why the expression levels of IFN‐γ, IL‐2 and IL‐17 mRNA were reduced to different degrees when treated with QOL, and it was speculated that the anti‐coccidiosis effect of QOL might not be realized by regulating the expression levels of IFN‐γ, IL‐2 and IL‐17 mRNA. Its anti‐coccidial effect may be achieved by inhibiting the formation of coccidial oocyst wall and reducing oocyst emissions by artemisinin, an effective component of QOL (Mo et al., 2014).

## 5 CONCLUSION

In conclusion, QOL has a positive anti‐coccidiosis effect. The anti‐coccidiosis index of the high‐dose was up to 173.08, and the efficacy evaluation is good. In addition, we found that the effect of QOL on cellular immunity of chickens artificially infected with coccidiosis is not obvious.

### PEER REVIEW

I would not like my name to appear with my report on Publons https://publons.com/publon/10.1002/vms3.922.

## CONFLICT OF INTEREST

We declare that we have no financial and personal relationships with other people or organizations that can inappropriately influence our work, there is no professional or other personal interest of any nature or kind in any product, service and/or company that could be construed as influencing the position presented in, or the review of, the manuscript entitled, ‘Effect of Qing Chang Oral Liquid on the Treatment of Artificially Infected Chicken Coccidiosis and the Cellular Immunity’.

## FUNDING STATEMENT

The experimental materials and reagents of this project are all funded by Chongqing Natural Science Foundation (Chongqing Natural Science Foundation Project (cstc2019jcyj‐msxmX0061).

## ETHICAL APPROVAL STATEMENT

This article does not contain any studies with human participants or animals performed by any of the authors. Informed consent was obtained from all individual participants included in the study.

## AUTHOR CONTRIBUTIONS

Yan Zhi‐qiang and Chen Qian‐lin designed experiments. Fu Li‐zhi and Fu Wen‐gui carried out experiments. Zheng Hua analysed experimental results. Tang Hong‐mei analysed sequencing data and developed analysis tools. Yan Zhi‐qiang, Chen Chun‐lin and Zhai Shao‐qin wrote the manuscript.

## Data Availability

The data used to support the findings of this study are included within the article.
